# Supplementary material for: Developing a Classification of Spinal Medical Devices: Has the Time Come? Review of the Literature and a Proposal for Spine Registries
Source: Bioengineering (Basel). 2025 Aug 8;12(8):853. doi: 10.3390/bioengineering12080853 (PMC12383846; doi:10.3390/bioengineering12080853)
Supplement: Supplementary file 1 [file bioengineering-12-00853-s001.zip › bioengineering-3724649-supplementary.pdf]

SUPPLEMENTARY MATERIALS

Developing a classification of spinal medical devices: has the time come? Review of the literature and a proposal for spine registries

Literature search strategy

The Italian Spine Registry Working Group (WG) decided to develop a literature review of the available online biomedical literature about the classification of spinal devices. Therefore, the WG discussed methodological search issues to define inclusion/exclusion criteria for the online literature search, suggested the appropriate keywords for building the search strategy and set criteria for getting articles. The online search covered the highly structured peer-reviewed databases PubMed/Medline, Biological Science Collection (ProQuest), Scopus (Elsevier), and Web of Science (Clarivate). The WG deemed it crucial not to miss any potentially relevant studies. Therefore, a sensible search strategy was set based on the main key question:

What and how many spinal device classifications exist and are currently used by spine registries worldwide?

Based on this key question, queries were set for each database to find articles on taxonomy and spinal registers with any study design, no publication date or language restrictions, based on the following keywords and text word: spinal device, taxonomy, classification, characteristics.

The present document presents the search strategies applied to the selected databases.

The search stopped on 6 May 2024.

PubMed/MEDline

(taxonomy [mh] OR classification* [tiab] OR classified [tiab] OR systematiz* [tiab] OR taxonomies [tiab] OR nomenclature [tiab] OR terminology [tiab])

AND

("spine device*" [tiab] OR "spine device*" [ot] OR "spinal device*" [tiab] OR "spinal device*" [ot] OR "spinal fixation" [tiab] OR "spinal fixation" [ot] OR "spine fixation" [tiab] OR "spine fixation" [ot] OR (("augmentation system*" [tiab]) OR (filler [tiab]) AND (spine OR spinal)) OR (implant* AND (discal [tiab] OR discal [ot] OR vertebr* [tiab] OR vertebr* [ot] OR spine OR disc* [tiab] OR discs [tiab] OR disc* [ot] OR discs [ot] OR cervical [tiab] OR cervical [ot] OR lumbar [tiab] OR lumbar [ot] OR dorsal* [tiab] OR dorsal* [ot] OR discal [tiab] OR discal [ot])) OR "spine surgery" [tiab] OR "spine surgery" [ot] OR "spinal surgery" [tiab] OR "spinal surgery" [ot]

Biological Science Collection (ProQuest)

(title(taxonomy OR nomenclature OR classification OR terminology)

AND

title(implant OR implantation OR device OR prosthesis OR prosthethic OR protheses))

AND

(subject(spine OR spinal OR vertebral OR vertebrae OR intervertebral OR discal OR disc OR hernia OR arthrodesis OR arthroplasty OR fusion))

Scopus (Elsevier)

(TITLE ((taxonomy OR nomenclature OR classification OR terminology))

AND

TITLE ((implant OR implantation OR device OR prosthesis OR prosthetic OR protheses))

AND

TITLE-ABS-KEY ((
spine OR spinal OR vertebral OR vertebrae OR intervertebral OR discal OR disc OR hernia OR arthrodesis OR arthroplasty OR fusion)))

AND

(LIMIT-TO (EXACTKEYWORD , "Human"))

Web of Science (Clarivate)

(--TS=(taxonomy OR nomenclature OR classification OR terminology)

AND

(--TS=("prosthesis implantation" OR implantable OR prostheses OR implants OR implanted OR "permanent device")

AND

(--TS=((spine OR spinal OR vertebral OR vertebrae OR intervertebral OR discal OR disc OR hernia OR arthrodesis OR arthroplasty OR fusion))--)

AND

LANGUAGE: (English OR French OR Italian OR Spanish)

Indexes=SCI-EXPANDED, SSCI, CPCI-S, CPCI-SSH, ESCI
